# Physical Activity Monitoring and Classification Using Machine Learning Techniques

**DOI:** 10.3390/life12081103

**Published:** 2022-07-22

**Authors:** Saeed Ali Alsareii, Muhammad Awais, Abdulrahman Manaa Alamri, Mansour Yousef AlAsmari, Muhammad Irfan, Nauman Aslam, Mohsin Raza

**Affiliations:** 1 Department of Surgery, College of Medicine, Najran University Saudi Arabia, Najran 61441, Saudi Arabia; manaa_880@hotmail.com (A.M.A.); dr.aboyousef@hotmail.com (M.Y.A.); 2 Department of Computer Science, Edge Hill University, St Helens Rd, Ormskirk L39 4QP, UK; razam@edgehill.ac.uk; 3 Electrical Engineering Department, College of Engineering, Najran University Saudi Arabia, Najran 61441, Saudi Arabia; miditta@nu.edu.sa; 4 Department of Computer and Information Sciences, Northumbria University, Newcastle upon Tyne NE1 8ST, UK; nauman.aslam@northumbria.ac.uk

**Keywords:** digital health, e-health, pandemic, physical activity, machine learning, performance evaluation

## Abstract

Physical activity plays an important role in controlling obesity and maintaining healthy living. It becomes increasingly important during a pandemic due to restrictions on outdoor activities. Tracking physical activities using miniature wearable sensors and state-of-the-art machine learning techniques can encourage healthy living and control obesity. This work focuses on introducing novel techniques to identify and log physical activities using machine learning techniques and wearable sensors. Physical activities performed in daily life are often unstructured and unplanned, and one activity or set of activities (sitting, standing) might be more frequent than others (walking, stairs up, stairs down). None of the existing activities classification systems have explored the impact of such class imbalance on the performance of machine learning classifiers. Therefore, the main aim of the study is to investigate the impact of class imbalance on the performance of machine learning classifiers and also to observe which classifier or set of classifiers is more sensitive to class imbalance than others. The study utilizes motion sensors’ data of 30 participants, recorded while performing a variety of daily life activities. Different training splits are used to introduce class imbalance which reveals the performance of the selected state-of-the-art algorithms with various degrees of imbalance. The findings suggest that the class imbalance plays a significant role in the performance of the system, and the underrepresentation of physical activity during the training stage significantly impacts the performance of machine learning classifiers.

## 1. Introduction

Regular physical activity plays a vital role in improving the health of individuals, whether it is a child under 5 or an elderly above 65. Physical activity has well-documented health benefits and can extensively improve the health and well-being of individuals and reduce the risks from noncommunicable diseases. Both moderate- and vigorous-intensity physical activity improve health. Physical inactivity increases the risk of noncommunicable disease mortality and puts inactive people at a 20–30% higher risk of death in comparison to physically active people [1]. Physical inactivity is among the leading factors which cause mortality and is estimated to contribute to 6% of worldwide deaths [2]. Therefore, World Health Organization (WHO) also recommends people of all ages indulge in physical activity and recommends the duration and intensity of physical activity for different age groups [1]. It has been noted that physical activity improves muscular and cardiorespiratory fitness, bone health and mental fitness while reducing the risk of heart diseases, diabetes, hypertension, obesity and fractures [1].

Physical activity and the promotion of healthy living can significantly lower the risks of non-communicable diseases. It also serves as the best remedy for obesity [3]. Obesity is one of the major chronic illnesses and increases the risk of developing many serious comorbidities, such as hypertension, sleep apnea, type 2 diabetes, depression, etc. [3]. Furthermore, obesity is becoming an increasingly prevalent issue. Obesity has become a global epidemic, with global stats suggesting nearly one-third of the world population is obese or overweight. Obesity has also added a significant burden to healthcare services, with nearly 10% of the medical costs in the US being spent on obesity-related issues. It also has been among the major causes of death in the US. Similarly, in Saudi Arabia, with 36% of the population being obese and 69% being categorized as overweight, nearly 20,000 lives are claimed to obesity each year. Therefore, under such circumstances, the provision of physical activity (PA) as a measure to control obesity has become increasingly important [4].

Obesity is one of the prevailing problems responsible for several health issues and medical conditions. Weight loss surgery, also referred to as bariatric or metabolic surgery, is one of the possible solutions for extremely overweight people. While the surgery can result in significant weight losses, it is still not termed a cure for obesity. Obesity is not a matter of concern only for the younger and older adults as it has become very common in children as well [5,6]. Therefore, suitable lifestyle changes should be introduced to avoid regaining weight. Patients who have undergone weight loss surgery need a balanced diet along with regular exercise once they have recovered from surgery. They also need to maintain a regular appointment schedule to keep everything in check. It is therefore important that a technology-driven framework for long-term support is developed to assist these patients in prolonging their healthy living choices and balancing exercise and diet accordingly. With the emergence of digital technologies, information and communications technology (ICT) solutions, machine intelligence and system analytics, post-surgery and long-term support can be efficiently managed with technology-driven solutions. This work primarily focuses on devising effective solutions for monitoring the physical activity levels of the patients in the post-surgery phase to maintain healthy living and discourage weight gain.

The increasing stress on the healthcare systems and the need to promote healthy living urge new measures to promote physical activities. The initial step in encouraging the physical activity is the ability to be able to quantify the physical activity into individual components of tangible impact. As such, physical activity classification can serve as a foundation by recording and transforming physical activities of an individual to give accurate quantification of a daily routine, thus encouraging active and healthy living. This highlights a clear need to develop feasible solutions to monitor the activities of daily living (ADLs) as a measure to avoid/overcome obesity.

Physical activities and exercise both serve as necessary measures for healthy living and maintaining healthy weights. Exercise is the subbranch of physical activity, and it is more structured, repetitive and planned with an intention to maintain or improve body fitness [7]. The promotion of physical activities is towards establishing and maintaining healthy living habits, such as walking to work, using stairs instead of lifts, use of muscles instead of motorized tools, etc. While promoting physical activities offer a more sustainable solution for staying active, it still needs to be quantized to give a better estimation of the efforts put in by the individuals and how these have impacted their healthy living. Quantifying the physical activities performed offers a means to relay the impact to the individuals as well as the medical staff to better evaluate the active status and suggest/intervene accordingly.

The physical activities are logged in several ways where questionnaires and direct observations are conventionally used. The logging of activities requires information on the type of activity performed, the duration for which it was performed and the intensity of the activity. An example could be walking, where the information about how much time is spent walking in a day/week, walking pace, etc. However, these are not as accurate and add additional time commitments from the observee and observer. Therefore, novel techniques are needed to use technology-driven solutions to log the type of activity performed, its duration and intensity.

The recent developments in the miniaturization of inertial sensors equipped with state-of-the-art processing and communication capabilities lay the foundations for the smart health and activity monitoring using machine learning techniques [8,9]. Wearable inertial measurement units (IMUs) use accelerometers and gyroscopes to measure acceleration and angular velocities to offer unobtrusive, reliable, and low-cost measurement of sensory data for physical activity classification. Single or multiple wearable IMUs can be placed on various body locations to classify daily life activities [10].

These small battery-operated wearable IMUs not only offer ease of use but are also equipped with transceivers to accumulate the vitals and activity data of patients to fog/cloud. The data accumulated at cloud or fog devices can be further processed using machine learning techniques [11,12] to identify the activity performed, its duration and intensity. While some existing works offer activity classification, however, there is still much room for improvement.

In [13], the authors proposed a solution for activity classification to identify strange behavior using support vector machines (SVM); however, it used surveillance videos instead of wearable sensors, and the focus of the work was security. Another similar study was carried out in [14], where abnormal behavior of a person was identified using pose estimation. Both these techniques, while detecting physical attributes, are still much further from the objectives of this work and use visual sensors/cameras instead of wearable devices.

In [15], the authors use the asymmetric 3D Convolutional Neural Networks for action recognition. The work was tested on the UCF-101 dataset, which combines actions from YouTube videos. While the claimed results were promising, the work was more tilted towards the general-purpose activity classification and use of visual sensing. Another work presented in [16] provides a unified framework for exploring multidimensional features in conjunction with body part models for pose estimation. A maximum entropy Markov model was used as a recognition engine which was claimed to have accurately detected body parts and recognized physical activity performed.

In [17], the authors used multimodal feature-level fusion for activity recognition. K-nearest neighbor and SVM were used for the classification of activities. As an input to the classification system, RGB camera, depth and inertial sensors data were used. While diversity was exploited, the camera usually conflicts with personal and security preferences and offers a limited field of view. Similarly, the depth sensor can also work only in a constrained field of view, which limits the scope of the work. In addition, the study was not focused on activities inspiring healthy living and controlling obesity.

In [18], the authors examined the relationship between physical activity and weight status. The performance of several machine learning techniques was evaluated on a largescale dataset. The objective of the study was to link physical activity with obesity. However, no sensory data were used to classify or log physical activities.

The existing literature and research studies use diverse techniques for activity classification with a wide scope of applications [18,19,20,21]. These applications range from security, autonomous transportation, expression evaluation, healthcare, etc. A relatively wide variety of sensors are also used, with some less suitable for the proposed work. While there are a variety of studies focusing on activity classification in healthcare using wearable IMUs [11,12,22,23,24,25], these focus on well-balanced data where all the physical activities performed are of equal samples. However, it is important to mention that in real life setting, physical activities (e.g., sitting, standing, walking, lying, stairs up, stairs down, etc.) are unstructured. Therefore, the natural occurrence and frequency of each activity cannot be controlled. This can lead to an imbalanced set of activities where certain classes of activities have more samples, data instances and sensory data than others [10,26]. Joana et al. [27] also found that underrepresented physical activities can affect the performance of the machine learning classifiers due to the availability of limited data at the training stage of the classifier. Therefore, it is important to not only study the impact of class imbalance on the performance of machine learning classifier when classifying physical activities but also investigate how such machine learning classifiers behave when more than one class of physical activities are imbalanced at their training stage. To the best of our knowledge, none of the existing studies have investigated the effect of multi class imbalance induced at the classifier training stage and its impact on the performance of physical activity classification. Moreover, the study also investigated a variety of machine learning classifiers to observe, which are more sensitive to class imbalance than others considering the overall performance of the physical activity classification system. Therefore, the work presented in this paper offers a unique contribution to evaluating physical activity to support healthcare professionals and medical staff in making correct interventions, maintaining diet and mandatory active living style for overweight and obese patients.

The main contributions of the paper are:

1. The paper compares several machine learning techniques to identify the best-suited activity classification techniques on a balanced dataset.
2. The physical activity dataset is intentionally skewed to introduce class imbalance and to evaluate the abilities of six well-known machine learning classifiers.
3. The proposed work compares the performance of the selected state-of-the-art machine learning algorithms with different training splits and various degrees of imbalance and identifies the best-suited machine learning techniques.

The rest of the paper is organized as follows: Section 2 presents the proposed system model including a data communication and activity classification framework, dataset used, feature computation, experimentation and implementation of machine learning algorithms. Research and discussion are covered in Section 3 and Section 4, respectively, whereas the concluding remarks and future directives are presented in Section 5.

## 2. System Model

**Figure 1:**
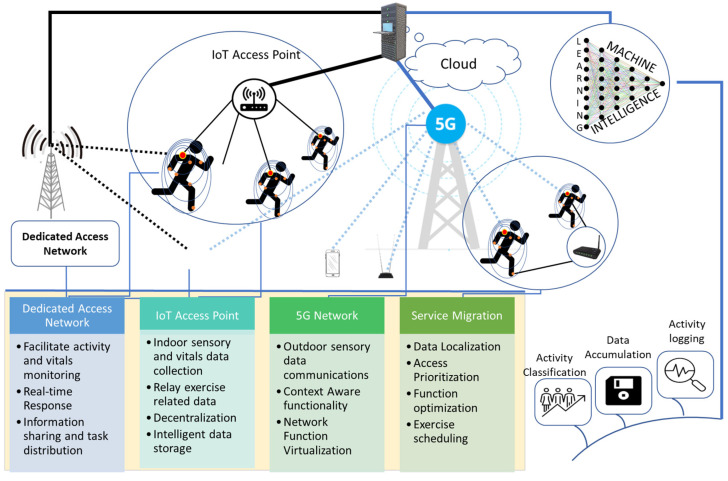
Post-surgery patient’s sensory data communications and activity classification framework.

In this work, a data communications and activity classification framework is presented, as shown in Figure 1. The proposed data communication and machine-learning-based activity classification framework lays out a communication infrastructure capable of sampling and relaying patients’ sensory data (vitals + activity-related data such as accelerometer readings etc.) over the internet to accumulate data from virtually infinite number of patients. It also proposes a machine-learning-based activity classification framework to process the accumulated patients’ data on the cloud and translate the sensory data into physical activities, thus maintaining the exercise/activity logs for each patient.

### 2.1. Wireless Communications Framework

The wireless communications framework intends to collect the data from different sensory elements mounted on the patient’s body. In addition, these data need to be relayed to the access points to be transferred over longer distances. Considering the applicability of the proposed framework in medical facilities, private indoor environments as well as public outdoor places, a multi-layer hybrid network is recommended. A modular approach must be followed to define a hybrid network to enable flexibility and scalability in network sizes. The two main operational blocks in the hybrid network framework consist of the body network and the Internet of Things (IoT) framework.

**Figure 2:**
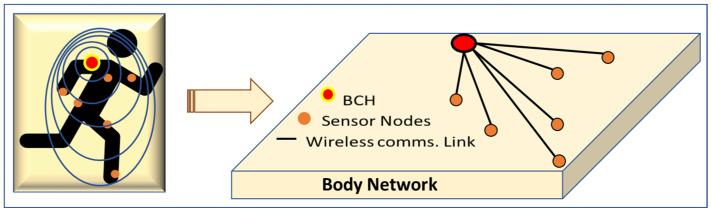
A representation of body network to collect sensory data from body-mounted sensors.

The sensory data collected from the potential multisensory agents on the body are communicated to the body communications hub (BCH) using the body network. A graphical representation of the body network is presented in Figure 2.

### 2.2. Machine Learning Paradigm for Physical Activity Classification

#### 2.2.1. Dataset

The study utilizes a publicly available dataset [28] to detect the activities of daily living (ADLs). The dataset is collected using a Galaxy S II smartphone by using its triaxial (3D) gyroscope and triaxial (3D) accelerometer sensors. The smartphone phone was mounted at a waist level to carry on data collection. Thirty subjects participated in the data collection experiment, aged from 19 to 48 years, and performed a variety of ADLs. The ADLs performed were lying, sitting, standing, walking upstairs, walking downstairs and normal walking. Data collection was conducted in a laboratory environment, and the participants were instructed to perform ADLs as naturally as possible. The sampling frequency of the accelerometer and gyroscope sensors was set to 50 Hz. The ground truth of the ADLs performed was maintained through visual observation.

#### 2.2.2. Feature Computation

Raw 3D accelerometer and gyroscope sensory signals obtained from the smartphone underwent several preprocessing and feature extraction steps to derive features that are fed to a machine learning algorithm later on to profile and classify ADLs.

The preprocessing steps involved are (i) low pass filtering using butter worth 3rd order filter at cutoff 20 Hz and (ii) median filtering. The acceleration signal is then divided into body acceleration and gravitational acceleration signals. To achieve this, a frequency of 0.3 Hz is used to separate the gravitational signals (<0.3 Hz) from the body acceleration signal (>0.3 Hz). Furthermore, jerk signals are derived from the acceleration signal and gyroscope signal by taking their derivates. The cadence is also derived from these signals. To analyze the frequency components, fast Fourier transform (FFT) is also computed to detect the trends and variations occurring in the frequency domain when different ADLs are performed. These derivations resulted in a total of 17 signals, including the original 3D gyroscope and 3D accelerometer signals. Further details about the feature extraction process can be found in [28].

The originals signals (3D accelerometer and 3D gyroscope) and the aforementioned derived signals are further processed using the windowing method to extract more features. The window length is set to 2.56 sec (128 samples of data) with 50% overlap (64 samples). Several statistical, time and frequency domains features are obtained from these derived signals, and each time, window of 128 samples is as follows: mean, standard deviation, median, signal magnitude area, maximum value, minimum value, angle between two signals, frequency domain band energy, skewness, kurtosis, average frequency component, maximum frequency component, correlation coefficient between signals, autoregressive correlation coefficients, interquartile range, sum of squares (energy) and band energy [28].

#### 2.2.3. Experiments

Several experiments have been conducted using a different split of the training and testing dataset to train the machine learning classifiers and to observe the performance of different machine learning classifiers in overbalanced and imbalanced datasets. Class imbalance is a critical issue in machine learning, and this often occurs when one or few classes are underrepresented (having fewer samples) than other classes. This often creates biases during the training stage of the machine learning algorithm, and the performance of underrepresented class or classes is highly affected by these imbalanced distributions.

Therefore, in this study, we also investigated the impact of imbalanced classes on the performance of the different machine learning classifiers by conducting different experiments. In addition, we also investigated which machine learning algorithms are relatively less sensitive to class imbalance or performed better than others.

The class distribution used in the first experiment or experiment 1 (E1) is presented in Table 1. The class distribution shown in Table 1 is the original class distribution obtained after the actual data collection. Each instance (number or sample) in Table 1 represents the number of time windows obtained for that particular class. Column 1 represents the activity type (walk, sit, stand, etc.), column 2 represents the total number of data instances obtained originally, column 3 represents the proportion of each activity class with respect to the total dataset, column 5 represents the train split or the number of time instances used to train the machine learning model and the last column represents the test split or the number instance used to test the performance of the machine learning algorithms. This original distribution or the balanced distribution of the ADLs in the train/test split is named experiment 1 (E1).

After designing experiment 1, six further experiments are conducted by inducing class imbalance in each class to observe the performance of the machine learning classifier in classifying different imbalanced ADLs. Table 2 represents the further six experiments conducted (from E2 to E7) in addition to E1 (please see Table 1).

It is important to note that training samples of the underrepresented classes are different in each of these experiments (from E1 to E7), while the test samples remain the same as per the original distribution presented in Table 1. This is due to the fact that the class imbalance added in the training samples may affect the performance of the machine learning classifier and the testing samples have no influence on the trained machine learning model.

#### 2.2.4. Machine Learning Algorithms Used

We implemented several machine learning algorithms on the dataset generated from 7 experiments and observed the performance of the different machine learning in classifying the ADLs in a balanced class distribution scenario (E1) and imbalanced class distribution scenarios (from E2 to E7).

The classifiers used in this study are support vector machine (SVM), Gradient boosting (GB) classifier, Extreme Gradient boosting (XGB) classifier, catboost (CB) classifier, AdaBoost classifier using decision tree (ADA-DT) and AdaBoost classifier using random forest (ADA-RF) [10,29,30,31,32,33]. The choice of classifiers is influenced by the fact that some of these are preferred due to their ensemble properties of combining the weak learners and improving the performance by collective or majority learning, while others, such as SVM, are widely used due to their hyper plane properties to create significant margin, thus achieving high performance [34,35].

All the simulations are performed in Python using its associated libraries. The parameters used to train these classifiers are as follows. The XGB parameters are maximum depth = 50, minimum child weight = 2, number of estimators = 100 and learning rate = 0.16. The GB parameters are objective function = multiclass, maximum depth= 50, learning rate = 0.1 and number of estimators = 100. The CB parameters are learning rate = 0.15, depth = 10 and loss function = Multi Class. The SVM parameters are kernel = linear, class weight = balanced and complexity = 1. The ADA(DT) parameters are Tree = Decision Tree Classifier with maximum depth = 10 and number of estimators = 100. The ADA(RF) parameters are Tree = Random Forest Classifier with number of estimators = 100, maximum features = auto and number of estimators = 100. Macro averaged F-score is used as a performance metric to compute the performance of the different classifiers in classifying the ADLs of daily living. The expression to calculate the F-score is expressed in Equation (1).

(1) $$F-score=\frac{2*TP_{C}}{2*TP_{C}+FP_{C}+FN_{C}}*100\;\;\;\;\;\;\;\;\;\;\;\;\;\;\;\;\;$$

where $TP_{C}$ represents true positive, $FP_{C}$ represents false positive, $FN_{C}$ represents false negative and the subscript c  represents the class it is computed for, such as sit, stand, waling, lie, etc.

## 3. Results

**Figure 3:**
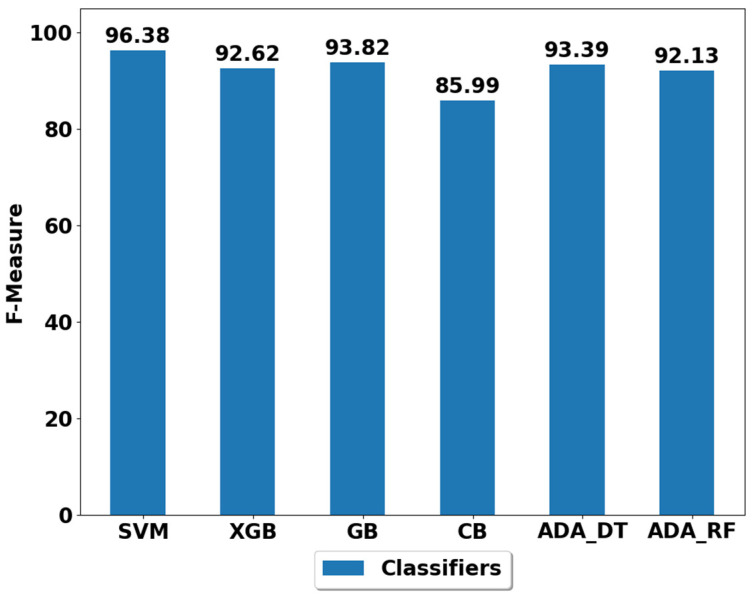
Performance analysis of classifiers using the train/test split in experiment 1 (E1).

**Figure 4:**
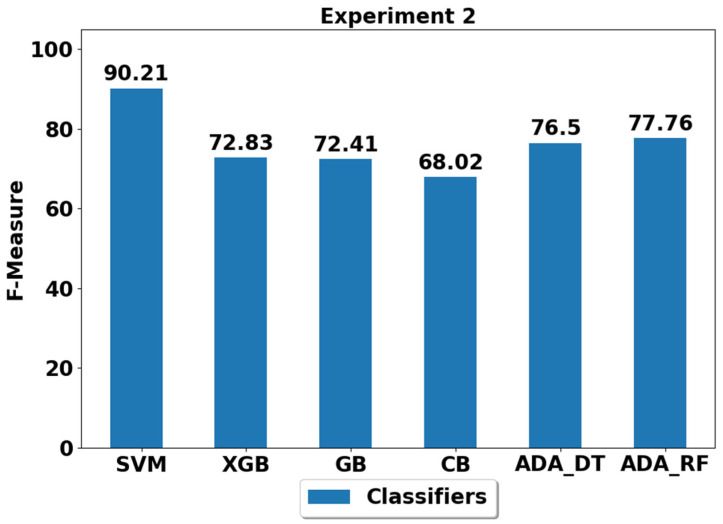
Performance analysis of classifiers using the train/test split in experiment 2 (E2).

**Figure 5:**
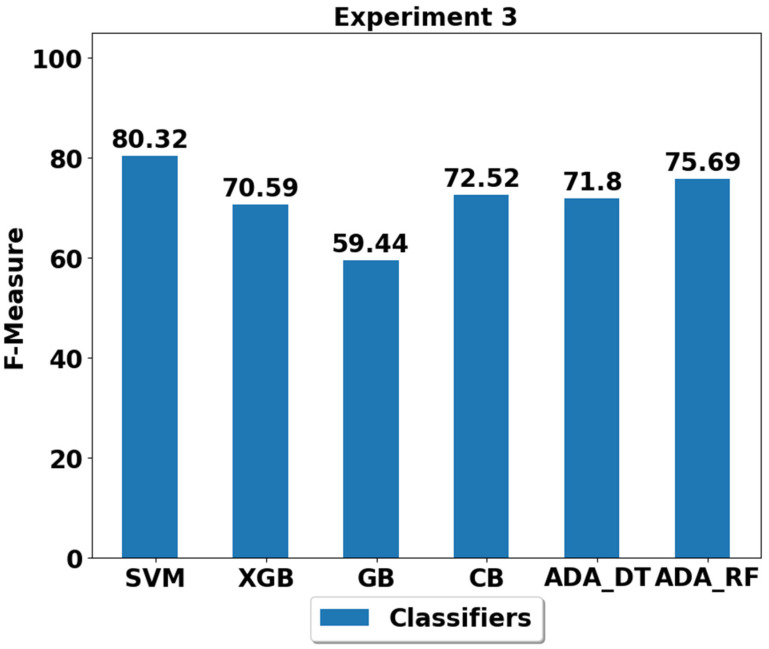
Performance analysis of classifiers using the train/test split in experiment 3 (E3).

**Figure 6:**
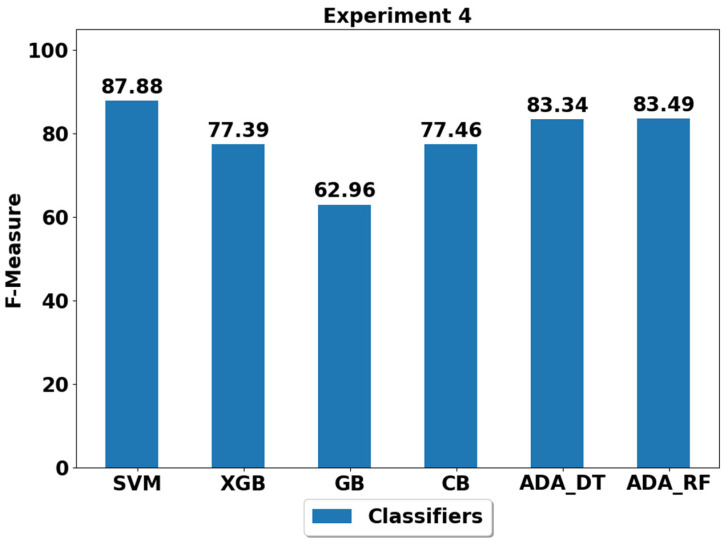
Performance analysis of classifiers using the train/test split in experiment 4 (E4).

**Figure 7:**
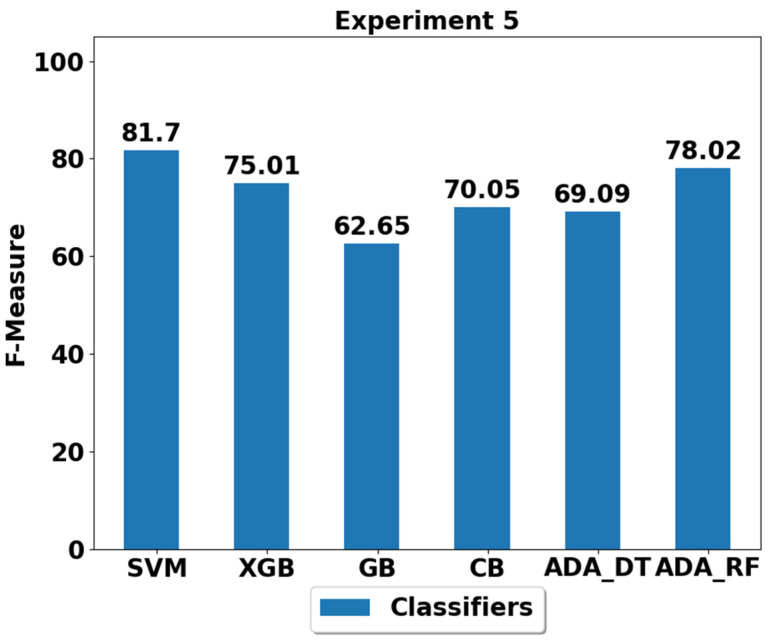
Performance analysis of classifiers using the train/test split in experiment 5 (E5).

**Figure 8:**
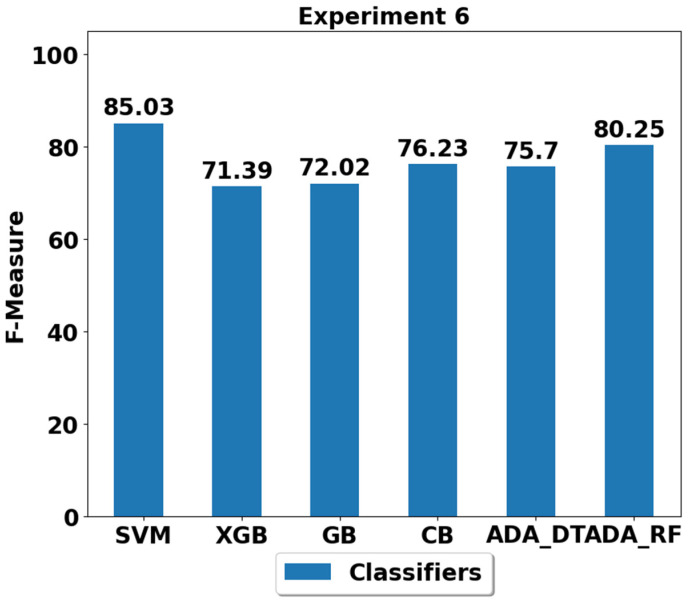
Performance analysis of classifiers using the train/test split in experiment 6 (E6).

**Figure 9:**
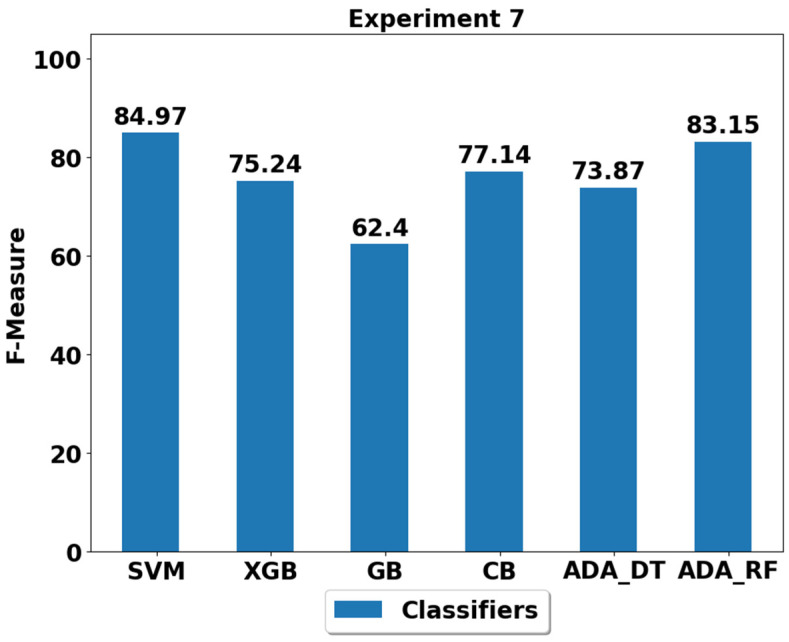
Performance analysis of classifiers using the train/test split in experiment 7 (E7).

The performance achieved using various machine learning classifiers for seven experiments (E1–E7) are presented in Figure 3, Figure 4, Figure 5, Figure 6, Figure 7, Figure 8 and Figure 9, and the respective performance by classes are presented in Table A1, Table A2, Table A3, Table A4, Table A5, Table A6 and Table A7 in Appendix A.

It is fairly evident from Figure 3 that all the classifiers have achieved the performance above 85% in classifying the ADLs (sitting, standing, walking, lying, walking upstairs and walking downstairs). These findings show the strength of the proposed machine-learning-based activity classification methods to classify ADLs. The best performer among all classifiers appeared to be SVM which achieved a performance of 96.38% (please see Figure 3). The SVM also outperformed the activity classification method proposed by Anguita et al. [28], thus confirming performance improvement when compared to the existing works. The second-best performer is GB, with the performance of 93.82%. All other classifiers also performed considerably well except the CB, whose performance is worst among all (85.99%). The detailed performance by class is visualized in Table A1. It is evident from Table A1 that most low-performing classifiers largely struggled in distinguishing between sitting and standing activities and struggled to distinguish between upstairs and downstairs walking. This could be due to the fact the smartphone is waist mounted during the data collection and the standing and sitting postures with respect to the accelerometer and gyroscope signals are relatively similar considering the smartphone orientation. The same is the case during upstairs and downstairs activities, which could make it hard for the classifiers to distinguish between different postures and locomotive activities. However, the SVM performed well in this scenario, and this could be due to the fact that SVM use high margin and hyperplanes to distinguish between different classes during the training stage, which assisted in better distinguishing these ADLs (sit vs. stand, upstairs vs. downstairs).

In experiment 2 (E2), only the walking class is imbalanced during the training stage with a total of 100 samples, while number of samples of all other classes remained the same as per the original or balanced distribution (please see Table 2). As expected and evident from Figure 4 and Table A2, most classifiers struggled in classifying the walking activity due to its low representation in the training stage. This suggests that class imbalance has serious consequences on the overall performance of the activity classification system and on the performance of the underrepresented/imbalanced class (es). Our findings suggest that the best performance of 90.21% is obtained using the SVM classifier, while none of the other classifiers are able to achieve the performance above 80%. This is an interesting finding and suggests the strength of SVM in handling class imbalance. The SVM inherently possess the properties of adaptive weighting, which provides more weight to the imbalanced classes and fewer weights to the over represented or balanced classes [10], thus improving classification performance.

In experiment 3 (E3), walking and walking upstairs are imbalanced classes at the training stage with a total of 100 samples each. The results in Figure 5 and Table A3 suggest that the best performer is still SVM in classifying the different ADLs with performance of around 80%, and the second best is ADA (RF) with performance of 75.69%.

In experiment 4 (E4), the underrepresented classes or imbalanced classes are walking, walking upstairs and walking downstairs. The best performance of 87.88% is achieved by the SVM classifier and the second best candidates are ADA (DT) and ADA (RF), with performance of above 83%, as shown in Figure 6 and Table A4. The worst performer is GB, with an F-score of 62.96%. It is important to note that the performance of all the classifiers is generally improved in E4 as compared to in E3. This could be due to the fact that only walking and walking upstairs are imbalanced in E3, while in E4, walking, walking upstairs and walking downstairs are imbalanced. This suggests that in E3, class sample mismatch between walking upstairs and walking downstairs could have more biased induced in training classifiers due to class imbalance in only walking upstairs class and not in the walking downstairs class. However, this has been reduced in E4 since both walking upstairs and walking downstairs are imbalanced with equal proportion when compared to other classes. Thus, giving equal opportunities to most of the classifiers to train properly.

In experiment 4 (E4), walking, walking upstairs, walking downstairs and sitting are underrepresented and imbalanced as compared to other majority classes. The results shown in Figure 7 and Table A5 suggest that the SVM again outperformed all the other classifiers with an F-score of 81.7%, and ADA (RF) is the second-best classifier with an F-score of 78.02%.

During experiment 6 (E6), all classes are underrepresented except the majority class represented class, which is lying. The performance analysis of the classifiers using the E6 train/test split is shown in Figure 8 and Table A6. All the classifiers are able to achieve the performance of above 70%. Similar to previous experiments’ results, SVM outperformed all the classifiers with performance of 85.03%, and the second-best performance was obtained by the ADA (RF) classifier.

In experiment 7 (E7), all the classes are balanced with equal samples; however, the samples are very low (100, please see Table 2) when compared to the original samples (around 500 for each class, please see Table 2) in E1. Lower number of training samples can influence the performance of the machine learning classifiers since supervised machine learning is all about feeding sufficient data to the classifiers. Therefore, fewer samples mean fewer training opportunities for the classifier to estimate and quantify the underlying trends from the data. The performances of the different classifiers using the E7 dataset are depicted in Figure 9 and Table A7. The SVM and ADA (RF) classifiers performed well with the performance of 84.97% and 83.15%, respectively, while the lowest performance of 62.4% was achieved by the GB classifier.

## 4. Discussion

The findings of the study are rather interesting and suggest the effect of class imbalance on system performance and how different classifiers behave when training classes are highly imbalanced. The SVM proved itself to be the best performance among all classifiers, and the second-best classifier is the ADA (RF) classifier. The possible rationale behind the high performance of the SVM in all the experiments could be due to the fact that it uses an adaptive weighting approach at the training stage [10]. This adaptive weighting reduced the bias induced at the training stage due to the class imbalance and underrepresentation and penalized the majority of classes with weighted samples. Moreover, the ADA (RF) uses a more sophisticated random-forest-based method to train, which could have been able to handle class imbalance to some extent.

The analysis of the class imbalanced datasets also suggested that most of the machine learning classifiers investigated in this work are sensitive to class balance except SVM, which is less sensitive to class imbalance due to its inherited property of adaptive weighting at the training stage to compensate for class imbalance up to some extent. The direction that can be opted in future works is to investigate the methods that can deal with class imbalance by performing a variety of data-handling techniques. These methods include synthetic minority over-sampling technique (SMOTE) [36], adaptive synthetic sampling technique (ADASYN) [37], under sampling and over sampling [38]. Therefore, such methods should be implemented on physical activity classification dataset collected in real life conditions with more natural settings. It is also worth mentioning that treating class imbalance can be harmful in some scenarios, as reported by Goorbergh et al. [39]. This is because treating class imbalance also depends on the type of classifier implemented, application domain and type of class imbalance dataset, as highlighted in [40].

Nevertheless, it is worth mentioning that the findings of the study are very encouraging and suggest that the proposed methods can obtain very high performance of above 96% in classifying the activities of daily living (sitting, standing, walking, lying, walking upstairs and waking downstairs). This provides the strength of the proposed physical activity classification system and its applicability in real life conditions. Promoting quality of life and tracking daily life activities are strongly correlated with obesity since active life patterns discourage sedentary behaviors and reduce the onset of several diseases (hypertension, diabetes, cardiovascular diseases), including obesity. Profiling such ADLs for a relatively longer duration (weeks, months, years, etc.) not only provides a detailed insight to individuals but also provides a detailed overview of the activity behaviors to the healthcare care practitioners, who can then tailor and customize the treatment to those suffering from obesity and other severe conditions.

The proposed physical activity classification system is applicable to a variety of different application scenarios in daily life conditions. Since the dataset used in this study utilizes the in-built motion sensors (accelerometer and gyroscope) of smartphones, there is no need for a separate sensing unit or equipment to acquire the activity patterns and retrieve sensory data. This sensory dataset acquired through smartphone can benefit from the on-device processing unit to compute the task requiring low computational power. Further processing can benefit from the scenario presented in Figure 1, where IoT assessment points can transmit the data to the cloud and storage units, where more sophisticated machine learning models can be implemented to classify the activity patterns. These activity patterns can then be profiled (e.g., 2% running, 10% walking, 20% sitting, 25% lying, 10% standing, 33% other sedentary or active activity over the day) and provide the distribution of activities performed by any individual over the course of a day, week, months and even years. This will not only benefit the general population to adopt a healthier lifestyle and well-being but also tracks the individuals with health issues such as obesity. The profiling of obese individuals with health disorders can then be linked with the healthcare services via IoT to track the activity patterns of individuals and to develop be-spoke exercise and therapy plans to effectively reduce obesity and to become healthy and active members of society. As the proposed system only used the smartphone for data gathering, its applicability in large-scale studies would not require resource-intensive equipment to track activity patterns. Moreover, such large-scale studies should be practiced in the future to develop big datasets in real life conditions and to train data-intensive deep learning classifiers for the efficient classification of daily life activities.

While the proposed research offers great to possibly deal with real life situations, there are certain limitations. One of such limitations is that it uses the dataset of only healthy individuals due to the unavailability of the sensory datasets collected from overweight individuals. Therefore, future works should focus on collecting and analyzing the dataset of only obese or overweight individuals to classify the activity patterns. It is important to mention that conducting longitudinal studies for overweight cohorts to record sensory data requires significant resources. This is one of the reasons why the publicly available dataset is used for the analysis and classification of physical activities in the present work. In future work, it would also be interesting to investigate how the deep-learning-based machine learning classifiers’ (such as convolutional neural network (CNN) [41], long-short term memory (LSTM) [42] or other deep learning classifiers’) behaves on the imbalanced dataset. In future research, a broad range of deep learning techniques will be evaluated for imbalanced dataset to investigate their performance. Moreover, cloud-based computing paradigms can be explored in the future to enable scalability and remote accessibility. The future work should also focus on reducing the impact of class imbalance on the classifier’s performance by implementing data-handling techniques such as over-sampling, under sampling, SMOTE, ADASYN, etc.

## 5. Conclusions

The study developed a novel physical activity classification system and investigated the impact of class imbalance on the performance of machine learning classifiers. The findings concluded that the proposed system is capable of classifying daily life activities such as sitting, standing, walking, lying, walking upstairs and walking downstairs with very high accuracy (above 96%). In addition, a thorough analysis of the impact of class imbalance on the performance of classifiers’ is also investigated. A number of experiments are conducted with class imbalance. The findings also suggested that the weighted SVM with penalized approach offered the best classification performance, followed by the ADA(RF) in most of the experiments. Out of the six classifiers evaluated, the SVM, with an overall performance of above 80% in all the class imbalance experiments, depicts its ability to deal with real life situations with certain types of activities being underrepresented.

## Figures and Tables

**Table 1:** Class distribution of different ADLs in experiment 1 (E1).

Activity Type	Total Dataset	Percentage (Total Dataset)	Train Split	Test Split
Walk	1722	16.72%	1226	496
Upstairs	1544	14.99%	1073	471
Downstairs	1406	13.65%	986	420
Sit	1777	17.25%	1286	491
Stand	1906	18.51%	1374	532
Lie	1944	18.88%	1407	537

**Table 2:** Class distribution of different ADLs in training samples during experiments 1–7 (E1, E2, E3, E4, E5, E6 and E7).

Activity Type	E1	E2	E3	E4	E5	E6	E7
	Train Split	Train Split	Train Split	Train Split	Train Split	Train Split	Train Split
Walk	1226	100	100	100	100	100	100
Upstairs	1073	1073	100	100	100	100	100
Downstairs	986	986	986	100	100	100	100
Sit	1286	1286	1286	1286	100	100	100
Stand	1374	1374	1374	1374	1374	100	100
Lie	1407	1407	1407	1407	1407	1407	100

## Appendix A

**Table A1:** Performance by class of different classifiers using the train/test split in Experiment 1 (E1).

Activity Type	SVM (%)	XGB (%)	GB (%)	CB (%)	ADA (DT) (%)	ADA (RF) (%)
Walk	97.62	94.09	95.19	86.74	95.65	93.42
Upstairs	96.78	90.66	92.44	86.37	91.91	89.10
Downstairs	98.08	93.97	94.49	81.03	92.37	89.82
Sit	92.26	87.45	89.55	80.36	89.36	89.78
Stand	93.55	89.57	91.26	81.42	91.04	90.64
Lie	100.00	100.00	100.00	100.00	100.00	100.00
Overall	96.38	92.62	93.82	85.99	93.39	92.13

**Table A2:** Performance by class of different classifiers using the train/test split in Experiment 2 (E2).

Activity Type	SVM (%)	XGB (%)	GB (%)	CB (%)	ADA (DT) (%)	ADA (RF) (%)
Walk	76.92	15.96	9.23	6.25	25.35	34.11
Upstairs	90.80	76.23	78.80	72.26	80.81	82.22
Downstairs	87.73	70.76	74.00	65.22	70.03	69.82
Sit	92.26	85.93	85.53	81.76	90.74	89.73
Stand	93.55	88.13	86.90	82.60	92.05	90.67
Lie	100.00	100.00	100.00	100.00	100.00	100.00
Overall	90.21	72.83	72.41	68.02	76.50	77.76

**Table A3:** Performance by class of different classifiers using the train/test split in Experiment 3 (E3).

Activity Type	SVM (%)	XGB (%)	GB (%)	CB (%)	ADA (DT) (%)	ADA (RF) (%)
Walk	81.92	51.04	13.83	44.41	47.15	60.22
Upstairs	49.68	39.11	27.09	65.26	47.36	53.82
Downstairs	64.55	59.53	61.23	62.82	56.04	59.89
Sit	92.28	85.99	84.05	80.81	89.36	89.66
Stand	93.47	87.87	70.42	81.84	90.88	90.55
Lie	100.00	100.00	100.00	100.00	100.00	100.00
Overall	80.32	70.59	59.44	72.52	71.80	75.69

**Table A4:** Performance by class of different classifiers using the train/test split in Experiment 4 (E4).

Activity Type	SVM (%)	XGB (%)	GB (%)	CB (%)	ADA (DT) (%)	ADA (RF) (%)
Walk	84.30	62.19	23.49	69.53	77.59	75.24
Upstairs	79.21	67.58	40.00	71.26	74.44	73.49
Downstairs	77.99	76.32	77.01	73.67	78.60	72.94
Sit	92.28	72.36	78.74	73.70	82.92	89.05
Stand	93.47	85.91	58.54	76.87	86.48	90.19
Lie	100.00	100.00	100.00	99.72	100.00	100.00
Overall	87.88	77.39	62.96	77.46	83.34	83.49

**Table A5:** Performance by class of different classifiers using the train/test split in Experiment 5 (E5).

Activity Type	SVM (%)	XGB (%)	GB (%)	CB (%)	ADA (DT) (%)	ADA (RF) (%)
Walk	84.30	71.29	35.79	60.89	65.68	78.91
Upstairs	79.35	65.12	45.05	72.67	42.90	72.75
Downstairs	77.99	75.60	78.09	68.01	67.74	73.81
Sit	67.29	61.67	61.06	46.13	61.02	63.20
Stand	81.23	77.69	55.93	77.30	77.17	79.48
Lie	100.00	98.71	100.00	95.30	100.00	100.00
Overall	81.70	75.01	62.65	70.05	69.09	78.02

**Table A6:** Performance by class of different classifiers using the train/test split in Experiment 6 (E6).

Activity Type	SVM (%)	XGB (%)	GB (%)	CB (%)	ADA (DT) (%)	ADA (RF) (%)
Walk	84.30	58.55	65.23	61.04	F-score	69.80
Upstairs	79.35	55.60	48.78	67.14	64.38	71.53
Downstairs	77.99	76.61	79.96	67.58	57.96	72.94
Sit	83.12	65.34	66.55	79.92	70.77	84.11
Stand	85.43	74.15	80.73	81.68	80.08	83.47
Lie	100.00	98.08	90.86	100.00	80.99	99.63
Overall	85.03	71.39	72.02	76.23	100.00	80.25

**Table A7:** Performance by class of different classifiers using the train/test split in Experiment 7 (E7).

Activity Type	SVM (%)	XGB (%)	GB (%)	CB (%)	ADA (DT) (%)	ADA (RF) (%)
Walk	84.30	63.61	21.72	71.23	59.02	83.08
Upstairs	79.35	56.58	43.16	62.70	53.74	76.52
Downstairs	77.99	77.26	75.66	72.36	70.94	76.15
Sit	82.88	73.37	75.18	81.34	79.68	81.57
Stand	85.27	80.70	58.96	78.95	80.30	81.60
Lie	100.00	99.91	99.72	96.23	99.53	100.00
Overall	84.97	75.24	62.40	77.14	73.87	83.15
